# Two decades of biomonitoring polar bear health in Greenland: a review

**DOI:** 10.1186/1751-0147-54-S1-S15

**Published:** 2012-02-24

**Authors:** Christian Sonne, Robert J Letcher, Thea Ø Bechshøft, Frank F Rigét, Derek CG Muir, Pall S Leifsson, Erik W Born, Lars Hyldstrup, Niladri Basu, Maja Kirkegaard, Rune Dietz

**Affiliations:** 1 Department of Bioscience, Faculty of Science and Technology, Aarhus University, Frederiksborgvej 399, PO Box 358, DK-4000 Roskilde, Denmark; 2 Ecotoxicology and Wildlife Health Division, Environment Canada, National Wildlife Research Centre, Carleton University, Ottawa, Ontario, K1A 0H3, Canada; 3 Aquatic Ecosystem Protection Research Division, Environment Canada, Burlington, Ontario, L7R 4A6, Canada; 4 Department of Veterinary Disease Biology, Faculty of Life Sciences, University of Copenhagen, Bülowsvej 17, DK-1870 Frederiksberg, Denmark; 5 Greenland Institute of Natural Resources, PO Box 570, DK-3900 Nuuk, Greenland, Denmark; 6 University Hospital of Hvidovre, Kettegaards Allé 30, DK-2650 Hvidovre, Denmark; 7 Department of Environmental Health Sciences, University of Michigan School of Public Health, Ann Arbor, MI, USA; 8 Danish Agency for Science, Technology and Innovation Bredgade 40 DK-1260 Copenhagen K, Denmark

## Abstract

**Summary:**

We present an overview of studies of anthropogenic pollutants in East Greenland polar bears over the period of 1999-2011. East Greenland polar bears are among the most polluted species, not just in the Arctic but globally, and represent an excellent biomonitoring species for levels and effects of global pollution in an apex predator. Therefore, an international multidisciplinary team joined to monitor and assess the patterns and concentrations of contaminants and their potential negative impact on polar bears. The review showed that East Greenland polar bears are exposed to a mix of chlorinated, brominated and fluorinated organic compounds as well as mercury which are all known to have endocrine, immune and organ-system toxic properties. For example, the concentrations of PCBs (polychlorinated biphenyls) in blubber ranged approximately 800-21,000 ng/g lw while mercury concentrations in liver and kidney ranged 0.1-50 μg/g ww. Regarding health endpoints, bone density seemed to decrease as a function of time and OHC (organohalogen compound) concentrations and further T-score for adult males indicated risk for osteoporosis. .The size of sexual organs decreased with increasing OHC concentrations. In the lower brain stem, mercury-associated decreases in NMDA-receptor levels and DNA-methylation was found The present review indicated that age was one of the major drivers for liver and renal lesions, although contaminants and infectious diseases may also play a role. Lesions in thyroid glands were most likely a result of infectious and genetic factors and probably, together with endocrine disrupting chemical (EDCs), the reason for disturbances/fluctuations in blood plasma thyroid hormone concentrations. Except for bone density reductions and neurological measures, all findings were supported by case-control studies of Greenland sledge dogs exposed long-term orally to similar combinations of contaminant concentrations. The studies of sledge dogs also indicated that the mixture of contaminants and fatty acids in the blubber of prey similar to that of polar bears induces cellular as well as humoral immune toxic changes. These controlled studies using model species for polar bears indicate that the correlative findings between health endpoint and contaminants in polar bears could be a cause-and-effect relationship. Physiologically based pharmacokinetic (PBPK) modelling showed that the risk quotients were ≥1 for ΣPCB, dieldrin and PFOS, which indicate an increased risk of prenatally reproductive pathology. In conclusion polar bears are susceptible to long-range transported chemicals that may have various adverse effects on multiple organ systems such as the reproductive and immune system.

## Background

The use of East Greenland polar bears (*Ursus maritimus*) as a biomonitoring key species for measuring exposure to OHCs (organohalogen compounds) and Hg (mercury) was initiated in Scoresby Sound in 1983 and is now the most comprehensive time trend studies of pollution on this species [1-7]. In addition, museum samples of skin and skulls collected since year 1892 have been included for Hg analysis and various patho-morphological and toxico-pathological density analyses [4,6-11].

The studies of adverse health effects of pollution were started in 1999 by the implementation of a biomonitoring health programme via AMAP (Arctic Monitoring and Assessment Programme) [12-14]. The reason for choosing polar bears, and for upgrading the research intensity, was because the East Greenland polar bear subpopulation is one of the most contaminated and that local Inuit people rely on this species as a food resource in addition to ringed seal (*Phoca hispida*) that plays a much greater role. Because polar bears reflect temporal trends and biological effects of contaminants they may also serve as a proxy for human health exposure and possible effects despite the fact that the physiology, metabolism, food and way of life of these two species differ fundamentally [e.g. [5,7,13-15]].

We present an overview of contaminant concentrations and potential adverse health effects from anthropogenic contaminants in polar bears during the period 1999-2010. The health effects include decrease in bone density, morphological changes in sexual organs, liver, kidney and thyroid glands, as well as potential neurological alterations and impacts on the immune and endocrine systems. Finally, some speculations about the synergistic effects of environmental stressors (e.g. decrease in sea ice) on polar bear health are presented.

## Levels of contaminants

Chlorinated legacy contaminants (PCBs and OC pesticides), brominated flame retardants (PBDEs), perfluoroalkyl contaminants (PFCs) and Hg have been analyzed in brain, adipose tissue, liver, kidney, blood and hair samples from East Greenland polar bears. The range of contaminant concentrations used in relation to health endpoints during the period 1999-2002 are seen in Table 1. Concentrations increase in the order: PBDEs<PFCs<PCBs<Hg and are in the concentration of having adverse health effects according to the international scientific literature [13,14,16,17].

**Table 1:** Concentrations of various contaminants [Mean (Min-Max, *n*] divided on tissues in East Greenland polar bears sampled 1999-2002. All data are in ng/g lw except for nPCBs, PCDDs and PCDFs (pg/g lw), PFCs (ng/g ww) and mercury (µg/g ww). Reworked from Sonne (2010).

	Subcutaneous adipose tissue	Liver	Kidney	Brain	Blood
Lipid-%	88	11	-	21	1.3
∑PCB	6,543 (897-20,407, *92*)	28,409 (12,836-67,664, *20*)	-	148-2,186 (*20*)	538-15,692 (*20*)
HCB	102 (2.4-785, *92*)	109 (*20*)	-	15 (*20*)	28 (*20*)
∑HCH	194 (13-818, *92*)	67 (*20*)	-	15 (*20*)	12-146 (*20*)
Dieldrin	204 (26-866, *92*)	4,900 (*20*)	-	-	-
∑Chlordane	1,414 (243-7,465, *92*)	37,400 (*20*)	-	62 (*20*)	531 (*20*)
∑DDT	436 (73-1,580, *92*)	<0.1-476 (*20*)	-	ND	12-1,769 (*20*)
∑PBDE	70 (22-192, *92*)	127-936 (*20*)	-	<0.5-36 (*20*)	38-146 (*20*)
∑PFC	-	1,056-8,010 (29)	-	-	-
∑*n*PCB	241 (125-442, *5*)	124 (114-148, *5*)	-	-	-
∑PCDD	10 (7-12, *5*)	20 (8-38, *5*)	-	-	-
∑PCDF	4 (3-5, *5*)	14 (10-18, *5*)	-	-	-
∑OH/MeSO_2_-PCB	90-3134 (*20*)	1,882-18,018 (*20*)	-	66-352 (*20*)	29,692-226,154 (*20*)
∑OH/MeSO_2_-PBDE	<0.3-39 (*20*)	<0.5 (*20*)	-	<0.5 (*20*)	<0.5-1,060 (*20*)
∑MeSO_2_-p,p’-DDE	9 (*20*)	482 (*20*)	-	-	769 (*20*)
Mercury	-	11 (1-36, *59*)	14 (1-50, *57*)	0.4 (0.1-0.9, *82*)	-

According to Dietz et al. [3-5,7] the concentrations of legacy chlorinated contaminants (PCBs and OC pesticides) have decreased and stabilized since 1990, while newer contaminants like PFCs and contemporary threats like Hg have increased. So, despite international regulations on all these groups of contaminants they persist and biomagnify in the environment, which results in a cocktail of toxic chemicals in the tissues of East Greenland polar bears.

## Skeletal system

Analyses on the skeletal system have exclusively focused on skulls. The reasons for this are because Natural History Museums have archived these since 1892 and because skulls are relatively easily obtained in connection with the subsistence hunt.

**Figure 1:**
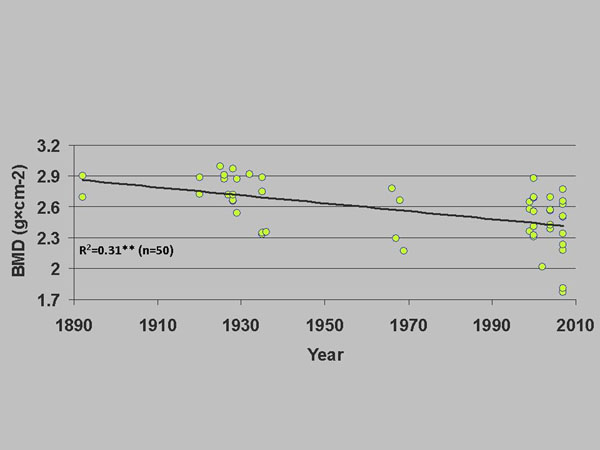
Bone mineral density (g×cm^-2^) as a function of sampling year in subadult East Greenland polar bears sampled 1892-2002. R^2^- and p-values from a full multiple regression model controlling for age. **: p < 0.01.

We examined skull bone mineral density (BMD) in 139 bears in the period 1892-2009 and after controlling for age and sex we found that BMD decreased significantly over time in subadults (Figure 1) and adult males but not in adult females (data not shown) [18]. That a similar decrease was not found in adult females was likely due to the fact that they increase bone density prior to denning in order to avoid demineralisation. Otherwise, the low mechano-transduction and limited food intake and high calcium flux via foetal development and lactation transfer during denning theoretically should lead to clinical osteoporosis [19].

**Figure 2:**
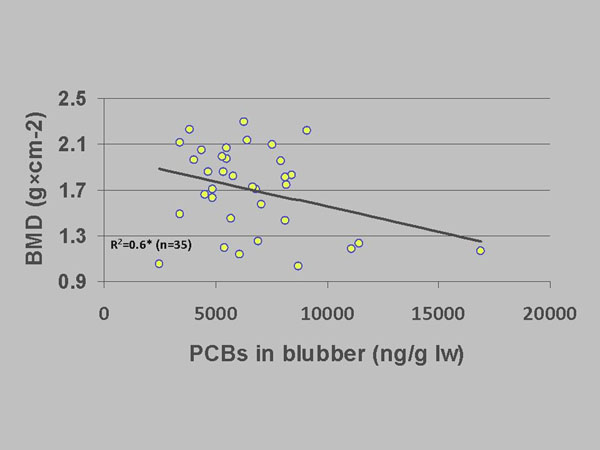
Bone mineral density (g×cm^-2^) as a function of adipose tissue PCBs (ng/g lw) in subadult East Greenland polar bears sampled 1999-2002. R^2^- and p-values from a full multiple regression model controlling for age. *: p < 0.05.

When correlating BMD with individual body burdens of OHCs in adipose tissue for individuals sampled during 1999-2002, significant inverse relationships were found in subadults (Figure 2) and adult males but not females [18]. Similar relationships were found for baculum BMD [20]. Furthermore, a calculation of T-score in males showed their risk of developing osteoporosis [14] as neuro-signalling may be influenced due to disruption of calcium homeostasis [21]. The exact pathways for these relationships are unknown; however, OHCs may disrupt brain and endocrine organ hormones as well as their transport proteins in polar bears as shown by multiple laboratory experiments [13,14,18]. During the study period 1892-2002 the pack sea ice in East Greenland has decreased [22]. Although undocumented, Sonne et al. [18] and Sonne [14] assumed that availability of polar bear prey (in East Greenland a variety of marine mammal prey other than ringed seals are available to polar bears) decreased simultaneous with the decrease in offshore sea ice and speculated whether this apparent decrease in sea ice may have led to increased energy expenditure and therefore indirectly served as a synergistic factor leading to reduced BMD.

## Reproductive organs

Studying impacts from OHCs optimally includes investigation of the reproductive tract as adverse effects on this organ-system have a direct impact on the population size [14].

**Figure 3:**
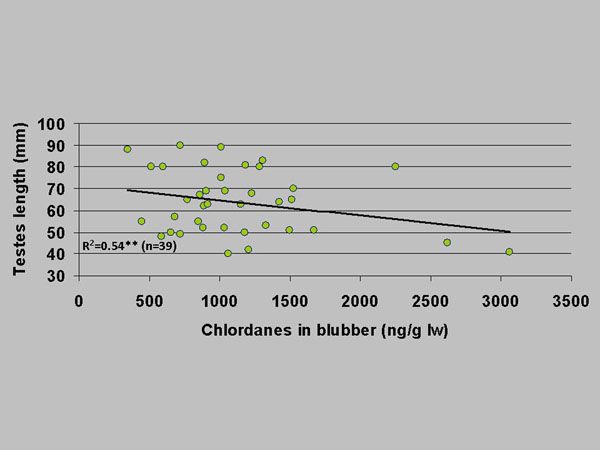
Testes length (mm) as a function of adipose tissue chlordane concentrations (ng/g lw) in 39 East Greenland polar bears sampled 1999-2002. R^2^- and p-values from a full multiple regression model controlling for age. **: p < 0.01.

We studied gross morphology and histopathology in sexual organs from 55 male and 44 female polar bears sampled 1999-2002. When controlling for age, the analyses showed that size of testicles, baculum/penis, and ovaries/female reproductive tract decreased as a function of increasing OHC concentrations in adipose tissue (Figure 3) [20]. In addition to this we found enlarged clitoris (megaclitoris) in two adults but ascribed this to chronic inflammation rather than OHC exposure [14,23]. In adult males we found chronic orchitis, atrophy, and fibrosis independently of season, all of which could be partly ascribed to OHC exposure and infectious pathogens as shown by high-dose, short-term controlled laboratory experiments [20]. The pathways for these are suspected to be disruptions of brain and endocrine organ hormones as well as their transport proteins [13,20]. It is not possible to fully estimate the impact from these disruptions and pathological conditions, however, it is likely that semen quality and quantity could be influenced, which may have fatal consequences for fecundity and maintenance of population size.

## Liver, kidneys and thyroid glands

Other key organs that are susceptible to endocrine disruption are the liver, kidneys, and various endocrine organs such and the thyroid glands and adrenals [13,14]. Liver (n=79), renal (n=75) and thyroid glands (n=20) were analyzed histologically in bears sampled 1999-2002. The evaluation, when not having a control group, is very difficult not least due to the effects from age and infectious diseases [14,24-28]. However, the various pathologies found in liver parenchyma and renal glomeruli, tubules and interstitium were similar to those found in controlled studies of Hg and OHCs in laboratory mammals and other OHC exposed wildlife [14,24-27]. The overall most important parameters determining liver and renal pathology was age while also some statistical relationships were found between pathology and different chlorinated and brominated groups of contaminants as well as the liver and renal toxic Hg concentrations.

No histological lesions were found in any of the 50 adrenals examined while pathology was found in 8 out of 20 examined thyroid glands [28]. None of the thyroid gland lesions including c-cell hyperplasia, interstitial fibrosis, and nodular hyperplasia were associated with age or gender, so environmental factors such as energetic stress and autoimmunity/genetic could be co-factors as well as OHCs. Such lesions may interfere with the hypothalamic–pituitary–thyroid (HPT) axis leading to endocrine disruptions having an impact on fecundity and foetal and neonatal development in East Greenland polar bears.

## Neuro-endocrine system

**Figure 4:**
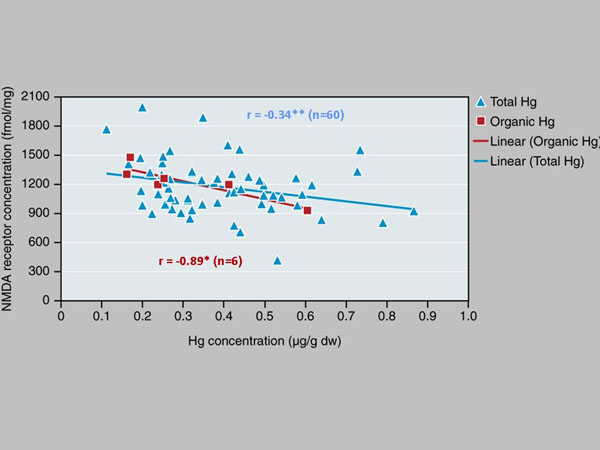
Correlations between NMDA-glutamate receptor levels and (A) Total Hg (blue) and (B) Organic Hg (MeHg; red) in the brain stem of 82 East Greenland polar bears sampled 1999-2002. Modified from Basu et al. [29]. *: p < 0.05.

During 1999-2002 we sampled the medulla oblongata from 82 specimens in order to analyze the concentrations of Hg, a known neuro-toxicant. The analyses showed that the concentrations of Hg were relatively low compared to liver and kidney burdens, as well as other species, probably due to high demethylation capacity of the liver, formation of Hg-selenium complexes, and fur as an efficient excretion route [29]. Despite the low Hg concentrations some inverse correlations of statistical significance were found. First, like in several other wildlife species [30,31], Hg-associated decreases in the levels of NMDA receptor were found. The NMDA-receptor facilitates the neurotransmission of glutamate and is important for learning and Hg (Figure 4). Second, DNA methylation seemed to decrease with increasing Hg concentrations indicating potential epigenetic alterations in gene expression [32].

Villanger et al. [33] analyzed the circulating full blood concentrations of TT3 and TT4 in 62 East Greenland polar bears. The reason for analyzing thyroid hormones is the fact that this endocrine system is known to be extremely susceptible to EDC exposure including biotransformed metabolites such as OH-PCBs [13,14,33]. The analyses showed that various biological parameters such as age, size, and condition affected the circulating concentrations while different organochlorine contaminants and bromated flame retardants had negative as well as positive effects on the concentrations of both TT3 and TT4. The conclusions were that these correlations indicate biological effects on the HPT axis from EDC exposure, which is also supported by various in vivo studies of laboratory mammals as well as in vitro studies of polar bears [13,14,33].

## Immune system

Very little has been published on the East Greenland polar bear immune system [13,14,34]. However, investigations on Svalbard bears have indicated immune toxic effects at OHC exposure concentrations similar to the East Greenland polar bear’s [13,14,35,36] and multiple studies in the laboratory and field show similar biological effects [13,14,37-39].

## Future challenges

**Figure 5:**
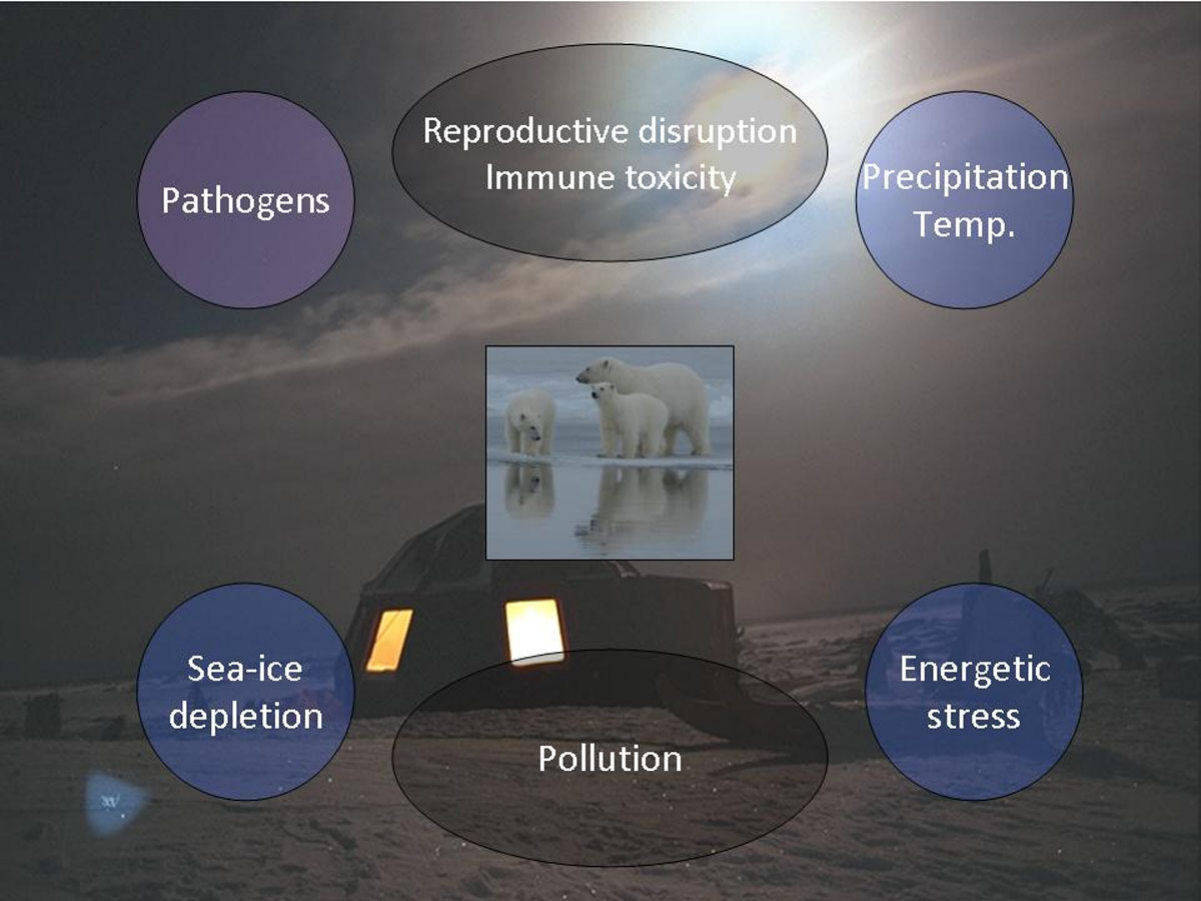
The multiple stressors in the Arctic that influences polar bear health. Oil and mineral activities, vessel shipment and subsistent hunt are not included.

As shown above, contaminant exposure is suggested to have potential health effects on various organ-systems in East Greenland polar bears. However, contaminants are not the only environmental stressor in East Greenland (Figure 5). Also global warming leading to decreased food access and negative energy balance may influence bear health via (sub)clinical impacts on immune functioning and reduced fecundity having an impact on populating size [13,14,40-42]. In addition to this global warming may also increase the infectious stress due to invasive micro pathogen and parasitic diseases [13,14]. The main challenge in the future is therefore to integrate the cumulative impact from these multiple stressors across temporal and spatial gradients by integrating empirical data and laboratory studies. The East Greenland polar bear seems an excellent biomonitoring organism for such.

## Conclusions

East Greenland polar bears are among the most contaminated species on our globe in spite of their remote Arctic habitat. This sub-population inhabits Arctic biotopes being constantly under pressure from global warming and associated environmental changes. Anthropogenic environmental contaminants seem to be a co-factor in various organ-system lesions in East Greenland polar bears. This includes reduced bone density and sexual and reproductive organ size, thereby having potential impacts on individual health and population maintenance. On top of this, also global warming seems to affect polar bears via negative energy balances, which may have consequences for fecundity and immune resistance. The main challenge in the future is to integrate the cumulative impact from these multiple stressors across temporal and spatial gradients by integrating empirical data and laboratory studies.

## References

[B1] Born EW Renzoni A Dietz R Total mercury in hair of polar bears (Ursus maritimus) from Greenland and Svalbard Polar Res 1991 9 113 120 10.1111/j.1751-8369.1991.tb00607.x

[B2] Dietz R Rigét FF Born EW Geographical differences of zinc, cadmium, mercury and selenium in polar bears (*Ursus maritimus*) from Greenland Sci Total Environ 2000 245 25 47 10.1016/S0048-9697(99)00431-3 10682354

[B3] Dietz R Rigét FF Sonne C Letcher RJ Born EW Muir DCG Seasonal and temporal trends in polychlorinated biphenyls and organochlorine pesticides in East Greenland polar bears (Ursus maritimus), 1990-2001 Sci Total Environ 2004 331 107 124 10.1016/j.scitotenv.2004.03.025 15325144

[B4] Dietz R Rigét FF Born EW Sonne C Grandjean P Kirkegaard M Olsen MT Asmund G Renzoni A Baagøe H Andreasen C Trends in mercury in hair of Greenlandic polar bears (Ursus maritimus) during 1892-2001 Environ Sci Technol 2006 40 1120 1125 10.1021/es051636z 16572764

[B5] Dietz R Bossi R Rigét FR Sonne C Born EW Increasing perfluorinated acids in East Greenland polar bears (Ursus maritimus) - a new toxic threat to the Arctic bears Environ Sci Technol 2008 42 2701 2707 10.1021/es7025938 18505019

[B6] Dietz R Outridge PM Hobson KA Anthropogenic contributions to mercury levels in present-day Arctic Animals Sci Total Environ 2009 407 6120 6131 10.1016/j.scitotenv.2009.08.036 19781740

[B7] Dietz R Born EW Rigét FF Sonne C Aubail A Basu N Temporal trends and future predictions of mercury concentrations in Northwest Greenland polar bear (Ursus maritimus) hair Environ Sci Technol 2011 45 1458 1465 10.1021/es1028734 21214235

[B8] Bechshøft TØ Wiig Ø Sonne C Rigét FF Dietz R Letcher RJ Muir DCG Temporal and spatial variation in metric asymmetry in skulls of polar bears (Ursus maritimus) from East Greenland and Svalbard Annal Zool Fenn 2008 45 15 31

[B9] Bechshøft TØ Sonne C Rigét FF Wiig Ø Dietz R Differences in growth, size and sexual dimorphism in skulls of East Greenland and Svalbard polar bears (Ursus maritimus) Polar Biol 2008 31 945 958 10.1007/s00300-008-0435-y

[B10] Bechshøft TØ Rigét FF Sonne C Wiig Ø Dietz R Letcher RJ Skull foramina asymmetry in East Greenland and Svalbard polar bears (Ursus maritimus) in relation to stressful environments Annal Zool Fenn 2009 46 181 192

[B11] Sonne C Rigét FF Dietz R Wiig Ø Kirkegaard M Born EW Gross skull pathology in East Greenland and Svalbard polar bears (Ursus maritimus) during 1892 to 2002 in relation to organohalogen pollution Sci Total Environ 2007 372 554 561 10.1016/j.scitotenv.2006.10.024 17156821

[B12] AMAP Assessment 2010: Mercury in the Arctic Arctic Monitoring and Assessment Programme: Oslo, Norway in press

[B13] Letcher RJ Bustnes JO Dietz R Jenssen BM Jørgensen EH Sonne C Verreault J Vijayan MM Gabrielsen GW Exposure and effects assessment of persistent organohalogen contaminants in Arctic wildlife and fish Sci Total Environ 2010 408 2995 3043 10.1016/j.scitotenv.2009.10.038 19910021

[B14] Sonne C Health effects from long-range transported contaminants in Arctic top predators: An integrated review based on studies of polar bears and relevant model species Environ Int 2010 36 461 491 10.1016/j.envint.2010.03.002 20398940

[B15] AMAP AMAP Assessment 2009: Human Health in the Arctic 2009 Arctic Monitoring and Assessment Programme (AMAP), Oslo, Norway xiv+256 http://www.amap.no

[B16] AMAP AMAP Assessment Report: Arctic Pollution Issues 1998 Arctic Monitoring and Assessment Programme (AMAP), Oslo, Norway xii+859 http://www.amap.no

[B17] AMAP AMAP Assessment 2002: Persistent Organic Pollutants in the Arctic 2004 Arctic Monitoring and Assessment Programme (AMAP), Oslo, Norway xvi+310 http://www.amap.no

[B18] Sonne C Dietz R Born EW Rigét FF Kirkegaard M Hyldstrup L Letcher RJ Muir DCG Is bone mineral composition disrupted by organochlorines in East Greenland polar bears (Ursus maritimus)? Environ Health Perspect 2004 112 1711 1716 10.1289/ehp.7293 15579418 PMC1253664

[B19] Lennox AR Goodship AE Polar bears (Ursus maritimus), the most evolutionary advanced hibernators, avoid significant bone loss during hibernation Comp Biochem Physiol Part A 2008 149 203 208 10.1016/j.cbpa.2007.11.012 18249018

[B20] Sonne C Leifsson PS Dietz R Born EW Letcher RJ Hyldstrup L Rigét FF Kirkegaard M Muir DCG Xenoendocrine pollutants may reduce size of sexual organs in East Greenland polar bears (Ursus maritimus) Environ Sci Technol 2006 40 5668 5674 10.1021/es060836n 17007124

[B21] Ganong WF Review of medical physiology 2005 22 USA: Appleton and Lange 928

[B22] Rosing-Asvid A The influence of climate variability on polar bear (*Ursus maritimus*) and ringed seal (*Pusa hispida*) population dynamics Can J Zool 2006 84 357 364 10.1139/z06-001

[B23] Sonne C Leifsson PS Dietz R Born EW Letcher RJ Kirkegaard M Muir DCG Andersen LW Rigét FF Hyldstrup L Enlarged clitoris in wild polar bears (Ursus maritimus) can be misdiagnosed as pseudohermaphroditism Sci Total Environ 2005 337 45 58 10.1016/j.scitotenv.2004.06.013 15626378

[B24] Sonne C Dietz R Leifsson PS Born EW Kirkegaard M Rigét FF Letcher RJ Muir DCG Hyldstrup L Do organohalogen contaminants contribute to liver histopathology in East Greenland polar bears (Ursus maritimus)? Environ Health Perspect 2005 113 1569 1574 10.1289/ehp.8038 16263513 PMC1310920

[B25] Sonne C Dietz R Leifsson PS Born EW Kirkegaard M Letcher RJ Muir DCG Rigét FF Hyldstrup L Are organohalogen contaminants a co-factor in the development of renal lesions in East Greenland polar bears (Ursus maritimus)? Environ Toxicol Chem 2006 25 1551 1557 10.1897/05-487R1.1 16764473

[B26] Sonne C Dietz R Leifsson PS Asmund G Born EW Kirkegaard M Are liver and renal lesions in East Greenland Polar Bears (Ursus maritimus) associated with high mercury levels? Environ Health 2007 6 11 10.1186/1476-069X-6-11 17439647 PMC1855925

[B27] Sonne C Bossi R Dietz R Leifsson PS Rigét FF Born EW The potential correlation between perfluorinated acids and liver morphology in East Greenland polar bears (Ursus maritimus) Toxicol Environ Chem 2008 90 275 283 10.1080/02772240701391629

[B28] Sonne C Leifsson PS Iburg T Dietz R Born EW Letcher RJ Kirkegaard M Thyroid gland lesions in organohalogen contaminated East Greenland polar bears (Ursus maritimus) Toxicol Environ Chem 2011 93 789 805 10.1080/02772248.2011.558669

[B29] Basu N Scheuhammer AM Sonne C Letcher RJ Dietz R Is dietary mercury of neurotoxicological concern to wild polar bears (Ursus maritimus)? Environ Toxicol Chem 2009 28 133 140 10.1897/08-251.1 18717617

[B30] Basu N Scheuhammer AM Rouvinen-Watt K Grochowina NM Evans RD O’Brien M Chan HM Decreased N-methyl-D-aspartic acid (NMDA) receptor levels are associated with mercury exposure in wild and captive mink Neurotoxicology 2007 28 587 593 10.1016/j.neuro.2006.12.007 17267038

[B31] Scheuhammer AM Basu N Burgess N Elliott JE Campbell GD Wayland M Champoux L Rodrigue J Relationships among mercury, selenium, and neurochemical parameters in common loons (*Gavia immer*) and bald eagles (*Haliaeetus leucocephalus*) Ecotoxicology 2008 17 93 101 10.1007/s10646-007-0170-0 17899374

[B32] Pilsner JR Lazarus AL Nam D Letcher RJ Scheuhammer T Sonne C Dietz R Basu N Mercury-associated DNA hypomethylation in polar bear brains via the LUminometric Methylation Assay (LUMA): A sensitive method to study epigenetics in wildlife Molecular Ecol 2010 19 307 314 10.1111/j.1365-294X.2009.04452.x 20002585

[B33] Villanger GD Munro Jenssen B Fjeldberg RR Letcher RJ Muir DCG Born EW Kirkegaard M Sonne C Dietz R Combined effects of mixtures of organohalogenated contaminants on thyroid hormones levels in polar bears Environ Int 2011 37 694 708 10.1016/j.envint.2011.01.012 21345491

[B34] Kirkegaard M Sonne C Leifsson PS Dietz R Born EW Letcher RJ Muir DCG Histology of selected immunological organs in polar bear (Ursus maritimus) from East Greenland in relation to levels of organohalogens Sci Total Environ 2005 341 119 132 10.1016/j.scitotenv.2004.09.034 15833246

[B35] Lie E Larsen HJS Larsen S Johansen GM Derocher AE Lunn NJ Norstrom RJ Wiig Ø Skaare JU Does high organochlorine (OC) exposure impair the resistance to infection in polar bears (Ursus maritimus)? Part I: Effect of OCs on the humoral immunity J Toxicol Environ Health A 2004 67 555 582 10.1080/15287390490425597 15129552

[B36] Lie E Larsen HJS Larsen S Johansen GM Derocher AE Lunn NJ Norstrom RJ Wiig Ø Skaare JU Does high organochlorine (OC) exposure impair the resistance to infection in polar bears (Ursus maritimus)? Part II: Possible effects of OCs on mitogen- and antigen-induced lymphocyte proliferation J Toxicol Environ Health A 2005 68 457 484 10.1080/15287390590903685 15799246

[B37] Sonne C Dietz R Larsen HJS Loft KE Kirkegaard M Letcher RJ Shahmiri S Møller P Impairment of cellular immunity in West Greenland sledge dogs (Canis familiaris) dietary exposed to polluted minke whale (Balaenoptera acutorostrata) blubber Environ Sci Technol 2006 40 2056 2062 10.1021/es052151d 16570636

[B38] Sonne C Fonfara S Dietz R Kirkegaard M Letcher RJ Shahmiri S Andersen S Møller P Multiple cytokine and acute phase protein gene transcription in West Greenland sledge dogs (Canis familiaris) dietary exposed to organic environmental pollutants Arch Environ Contam Toxicol 2007 53 110 118 10.1007/s00244-006-0135-y 17396211

[B39] Sonne C Larsen HJS Kirkegaard M Letcher RJ Dietz R Trans-generational and neonatal immune suppression in West Greenland sledge dogs (Canis familiaris) exposed to organohalogenated environmental contaminants Sci Total Environ 2010 408 5801 5807 10.1016/j.scitotenv.2010.07.076 20832100

[B40] Jenssen BM Endocrine-disrupting chemicals and climate change: a worst-case combination for Arctic marine mammals and seabirds? Environ Health Perspect 2006 114 76 80 16818250 10.1289/ehp.8057 PMC1874189

[B41] Molnár PK Derocher AE Thiemann GW Lewis MA Predicting survival, reproduction and abundance of polar bears under climate change Biol Conserv 2010 143 1612 1622 10.1016/j.biocon.2010.04.004

[B42] Molnár PK Derocher AE Klanjscek T Lewis MA Predicting climate change impacts on polar bear litter size Nature Comm 2011 2 186 DOI: 10.1038/ncomms1183 10.1038/ncomms1183 PMC3105343 21304515

